# Quantitative analysis of *Mycobacterium avium* subsp*. hominissuis* proteome in response to antibiotics and during exposure to different environmental conditions

**DOI:** 10.1186/s12014-019-9260-2

**Published:** 2019-11-13

**Authors:** Rajoana Rojony, Matthew Martin, Anaamika Campeau, Jacob M. Wozniak, David J. Gonzalez, Pankaj Jaiswal, L. Danelishvili, Luiz E. Bermudez

**Affiliations:** 1 Department of Biomedical Sciences, Carlson College of Veterinary Medicine, Oregon State University, Corvallis, USA; 2 Department of Botany and Plant Pathology, College of Agricultural Sciences, Oregon State University, Corvallis, USA; 3 Department of Microbiology, College of Sciences, Oregon State University, Corvallis, USA; 4 Department of Pharmacology, School of Medicine, Skaggs School of Pharmacy and Pharmaceutical Sciences, University of California San Diego, San Diego, USA

**Keywords:** *M. avium* proteomics, Amikacin, Clarithromycin, Macrophage, Biofilm, Anaerobic condition, Antibiotic susceptibility, Tolerance

## Abstract

*Mycobacterium avium* subsp. *hominissuis* (MAH) belongs to the clinically important non-tuberculous mycobacterial group that infects immunocompromised patients and individuals with underling lung conditions. The need for prolonged therapy is a major challenge of MAH treatment, influencing the development of persistent and drug-resistant infections. The reason why bactericidal drugs take several months to eliminate MAH is unknown. To investigate MAH proteome remodeling under aerobic, anaerobic and biofilm conditions (as it is encountered in patient lungs) and identify metabolic changes potentially associated with bacterial persistent state, we performed the relative protein quantitative analysis using Tandem Mass Tag Mass Spectrometry sequencing. MAH was exposed to amikacin (4 μg/ml) and clarithromycin (16 μg/ml) under aerobic, anaerobic or biofilm condition for 24 h and the response was compared with bacterial proteomics of the corresponding conditions. Overall, 4000 proteins were identified out of 5313 MAH proteome of across all experimental groups. Numerous sets of de novo synthesized proteins belonging to metabolic pathways not evidenced in aerobic condition were found commonly enriched in both anaerobic and biofilm conditions, including pantothenate and CoA biosynthesis, glycerolipid metabolism, nitrogen metabolism and chloroalkene degradation, known to be associated with bacterial tolerance in *M. tuberculosis*. The common pathways observed in anaerobic and biofilm conditions following drug treatments were peptidoglycan biosynthesis, glycerophospholipid metabolism and protein export. The LprB lipoprotein, highly synthesized in MAH biofilms during drug treatments and shown to be essential for *M. tuberculosis* virulence and survival in vivo, was selected and overexpressed in MAH. Results demonstrate that LprB is secreted in MAH biofilms and the overexpression clone is more tolerant to antimicrobials than the wild-type strain. Our study identified promising metabolic pathways that can be targeted to prevent the bacterial tolerance mechanism and, subsequently, reduce the length of MAH therapy.

## Introduction

*Mycobacterium avium* subsp. *hominissuis* (MAH), ubiquitously found in the environment, is an opportunistic pathogen associated with infections in human and other mammals [1]. Individuals with an underlying respiratory disease such as chronic obstructive pulmonary disease as well as cystic fibrosis are predisposed to MAH infection. MAH can also infect patients with immunosuppressed conditions and apparently healthy persons [2]. It is clear that the number of patients with nontuberculous mycobacterial (NTM) lung infections are in the rise in recent years [3].

The treatment of MAH infection, in general, relies on a macrolide-based (clarithromycin or azithromycin) therapy and includes ethambutol and rifampin or rifabutin [4, 5]; however, aminoglycosides (amikacin) are prescribed in advanced cases of infection and in MAH disease associated with a cavitary lesion [5]. Unfortunately, treatment of MAH cavitary lesions with a macrolide and an aminoglycoside results many times in unsatisfactory outcome [3, 6]. The main challenge of treating MAH patients is the inability to rapidly eliminate the infection; even when bactericidal concentrations of compounds are employed, resulting in use of the prolonged treatment. Although, the required extended period of therapy and multidrug treatment result in more favorable outcome for many patients, usually it eradicates MAH infection in only 40% to 60% of individuals [3, 7]. The explanation for an incomplete response to therapy is that the bacterium enters a nonreplicating persistent state, becoming tolerant to antibiotics. A low metabolic state during stress conditions is a common response observed in MAH. Evidence suggests that starvation or limitation in nutrients, low pH and lack of the oxygen induce a nonreplicating state in vitro characterized by modification of the metabolic state of the pathogen [8]. MAH can survive rapid shifts in and out of low oxygen condition for prolonged periods of time, by altering their metabolism from aerobic to anaerobic [9]. In addition, the phenotypic remodeling of MAH during biofilm formation facilitates development of bacterial tolerance to antibiotics. Biofilm structure, on the other hand, prevents the optimal penetration of antibiotics and interferes with drug killing mechanisms [10, 11].

Novel treatment approaches are urgently needed against MAH. There is a gap, however, in knowledge on MAH pathogenicity strategies and phenotypic changes associated with bacterial tolerance and survival following exposure with antibiotics. In addition, MAH is exposed to different environments encountered by the pathogen in the lung tissue and intracellularly, such as biofilm and anaerobic conditions.

To gain insights into MAH global proteome remodeling under aerobic, anaerobic and biofilm conditions, and determine the pathogen response in each condition following exposure to bactericidal concentrations of effective antimicrobials, we carried out proteomic study and identified metabolic changes promoting MAH tolerance in environments that the pathogen encounters within host cells. This study identifies several novel targets that potentially may contribute to rapid killing of MAH.

## Materials and methods

### Mycobacteria strains and culture conditions

*Mycobacterium avium* subsp. *hominissuis* 104 (MAH 104) is a virulent strain isolated from the blood of an AIDS patient and can cause pulmonary infections in mice [12]. MAH 104 was cultured onto 7H10 Middlebrook agar or in 7H9 Middlebrook broth (Difco Laboratories, Detroit, MI) supplemented with 10% oleic acid, albumin, dextrose, and catalase (OADC, Hardy Diagnostics, Santa Maria, CA). MAH was incubated at 37 °C until mid-log phase of growth (around 7 days). To prepare the inoculum, the bacterial suspension was adjusted to McFarland standard 0.5 (approximately 1.5 × 10^8^), and exact inoculum was confirmed by colony forming units (CFUs) where bacterial serial dilutions were plated on 7H10 agar.

### Antimicrobial reagents and susceptibility testing

Amikacin (AMK) was purchased from Sigma-Aldrich and clarithromycin (CLA) from TCI AMERICA. Amikacin and clarithromycin were solubilized in water and acetone, respectively. Susceptibility to drug concentrations in the range of 0.065–128 μg/ml were performed by the microdilution method in Hank’s Balanced Salt Solution (HBSS). Briefly, 3 × 10^5^ bacteria/ml were cultured in 7H9 Middlebrook broth supplemented with or without compounds and grown in a shaker at 37 °C for 7 days. Minimal Inhibitory Concentrations (MICs) were visually determined at day 7. Bactericidal concentrations were established by centrifuging tubes at 3500 rpm for 30 min and plating serially diluted bacterial pellets on 7H10 agar. Plates were incubated at 37 °C for 10 days. The drug concentration that inhibit 99.9% bacterial growth was considered as the bactericidal concentration.

### MAH killing kinetics in vitro

Approximately 3 × 10^5^ bacteria were inoculated into 3 ml of 7H9 broth containing either AMK 4 μg/ml, 16 μg/ml CLA or no antibiotic. Samples were incubated under 200 rpm agitation at 37 °C and, after 24 h, 48 h, 72 h and 96 h exposure, centrifuged for 30 min at 3500 rpm, serially diluted and plated onto 7H10 agar plates for CFU determination. To establish the MAH killing dynamics under an anaerobic condition, a suspension of 3 × 10^5^ bacteria were inoculated into 3 ml of 7H9 broth, with and without antibiotics, and tubes were placed into anaerobic jars (BD BBL™ GasPak™ Jar). The anaerobic BD BBL™ GasPak™ and CO_2_ indicators were inserted inside the jar and the grease was applied over the rim to seal the Jar tightly. The anaerobic jars (one jar for each time point) were kept in the shaker incubator at 37 °C with gentle agitation (30 rpm). At selected time points, the jar was opened, tubes were removed and centrifuged at 3500 rpm for 30 min. Pellet was resuspended in PBS, serially diluted and plated into 7H10-agar plate to CFU/ml counts. In addition, the killing kinetic was determined under the biofilm condition as well. Two hundred microliter of 3 × 10^6^ bacterial inoculum prepared in HBSS was distributed into 96-well tissue culture plates, covered with a cellophane membrane and kept at 25 °C for 7 days. After 7 days, the entire supernatant was removed and replenished with fresh HBSS containing bactericidal concentration of antibiotics, or lack of antibiotics. At selected time points, the biofilms were disrupted to obtain even suspensions and plated for viable bacteria counts. The experiment was carried out in duplicate and repeated three independent times.

### MAH killing kinetics in human macrophages

Human THP-1 cell line (TIB-202) was purchased from the American Type Culture Collection (Manassas, VA). Cells were cultured in RPMI-1640 medium supplemented with heat-inactivated 10% fetal bovine serum (FBS, Gemini Bio-products, Sacramento, CA), l-glutamine, and 25 mM HEPES (Corning, Manassas, VA) at 37 °C with 5% CO_2_ and maintained in 75 cm^2^ tissue culture flasks. THP-1 monocytes were differentiated into macrophages using 10 ng/ml of phorbol 12-myristate 13-acetate (PMA, Sigma-Aldrich). Intracellular antibiotic killing assays were performed as previously described with minor modifications [13]. Briefly, PMA treated THP-1 cells were seeded into 48-well plates at 90 to 100% confluence (3 × 10^5^/well), matured and 24 h latter monolayers were replenished with fresh medium. Monolayers were allowed to rest for an additional 48 h, and then infected with MAH 104 for 2 h at a multiplicity of infection (MOI) of 10. Extracellular bacteria were subsequently removed by both washing three times with HBSS and treating monolayers with 400 μg/ml amikacin for 1 h. Infected monolayers were treated with either AMK 4 μg/ml, 16 μg/ml CLA or no antibiotic as a control. THP-1 cells were replenished with new media containing antibiotics, or no antibiotic, every other day. Cells were lysed at 2 h (baseline), day 1, day 3, day 5 and day 7 followed by plating on 7H10 agar plates to determine the number of viable intracellular bacteria. For macrophage infection and antibiotic killing assay with MAH104 pre-incubated under anaerobic condition, bacterial suspension in HBSS was kept in the anaerobic chamber for 24 h at 25 °C, and then used as an inoculum to infect THP-1 cells with MOI of 10. To express the biofilm phenotype, we formed MAH biofilms for 7 days and then dispersed for the inoculum preparation as described above. Cells were replenished with fresh media containing antibiotics or no antibiotic every other day and lysed at 2 h (baseline), day 1, day 3, day 5 and day 7 to determine the number of viable intracellular bacteria.

### Sample preparation for proteomic sequencing

Approximately, 1 × 10^8^ *M. avium* 104 cells/ml of were inoculated in 10 ml of 7H9 culture media in the 25 cm^2^ tissue culture flasks supplemented with bactericidal concentrations of AMK or CLA for 24 h. For the aerobic condition, tubes were kept in the shaker at 37 °C and for the anaerobic condition samples were placed into anaerobic jar at 37 °C. In addition, biofilms were formed for 7 days in 10 ml HBSS using 1 × 10^8^ cells/ml inoculum in the 25 cm^2^ tissue culture flasks. After, the supernatant was gently removed and replenished with the fresh HBSS containing antibiotics for additional 24 h. Next, samples were centrifuged at 3500 rpm for 20 min at 4 °C, pellets were washed once with HBSS and resuspended in 3% SDS containing EDTA-free Protease Inhibitor Cocktail (Sigma-Aldrich). Bacterial cells were mechanically disrupted through bead-beating, cleared through microcentrifugation at 15,000 rpm for 10 min and filtration using the 0.22 µm syringe filters. Total protein concentrations were measured using Thermo Scientific NanoDrop machine.

### Tandem Mass Tag (TMT) labeling and Liquid chromatography–mass spectrometry (LC–MS)

Precleared lysates were immersed in equal volumes of 8 M urea and a lysis buffer containing 75 mM NaCl, 3% sodium dodecyl sulfate (SDS), 1 mM sodium fluoride, 1 mM beta-glycerophosphate, 1 mM sodium orthovanadate, 10 mM sodium pyrophosphate, 1 mM phenylmethylsulfonyl fluoride, 1× cOmplete EDTA-free protease inhibitor cocktail (Roche), and 50 mM HEPES (Sigma), pH 8.5. Samples were subjected to pulsed probe sonication to ensure complete cell lysis. A pulse protocol of 15 s “on” at 20% amplitude was alternated with 15 s periods of rest three times. Disulfide bonds were reduced in 5 mM of dithiothreitol (DTT) for 30 min at 56 °C. Reduced cysteine residues were alkylated in 15 mM of iodoacetamide (IAA) for 20 min in a darkened environment at room temperature. The alkylation reaction was subsequently quenched by the addition of the original added volume of DTT and incubation of the solution in a darkened environment at room temperature for 15 min [14].

Protein was precipitated by adding one quarter of the total sample volume of trichloroacetic acid (TCA) to the sample solution. Samples were vortexed and incubated on ice for 10 min before being subjected to centrifugation at 14,000 rpm for an additional 2 min. The resultant supernatant was removed and samples kept on ice for the subsequent wash steps. Samples were washed in 300 µl of ice-cold acetone and subjected to centrifugation at 14,000 rpm for 2 min. The supernatant was removed and the acetone wash step repeated. After removal of the second acetone wash supernatant, samples were dried on a 56 °C heating block. Samples were resuspended in 300 µl of a solution of 1 M urea and 50 mM HEPES, pH 8.5. Resuspended samples were subjected to 5 min of vortex-agitated and 5 min of water bath sonication. Samples were then subjected to a two-step digestion [15]. First, 3 µg of sequencing-grade endoproteinase LysC was added and samples were incubated on a shaker at room temperature overnight. Second, 2.8 µg of sequencing-grade trypsin was added to the samples and were incubated at 37 °C for 6 h. Samples were acidified by the addition of 20 µl of 10% trifluoroacetic acid and were desalted on C18 columns using previously-described methods [16]. Samples were lyophilized at this stage.

Lyophilized samples were immersed in a solution of 50% acetonitrile and 5% formic acid prior to quantification. Peptide quantification was performed using the Pierce Quantitative Colorimetric Peptide Assay (Thermo). 50 µg of each sample was separated for tandem mass tag (TMT) labeling [17, 18]. An internal standard containing an equal mass of each sample was prepared, and 50 µg of the standard was separated per intended set of 10 TMT labels. Sample aliquots designated for further analysis were lyophilized.

Lyophilized samples were resuspended in 50 µl of a solution of 30% anhydrous acetonitrile and 200 mM of HEPES, pH = 8.5. TMT labels were resuspended in 40 µl of anhydrous acetonitrile and subjected to vigorous shaking for 5 min. A TMT label assignment scheme was generated using two core principles: first, no two experimental replicates were assigned to the same label, and second, each experimental condition was represented in each set of 10 labels. Eight µL of each label was added to the designated sample, and the labeling reaction was allowed to proceed at room temperature for 1 h. Reaction quenching was performed by the addition of 9 µl of a solution of 5% hydroxylamine and room temperature incubation for 15 min. Reactions were acidified through the addition of 50 µl of a solution of 1% TFA. Samples assigned within each set of 10 labels were mixed, and each resulting mixture was desalted on C18 columns using the same methods as above.

Multiplexed samples were subjected to basic reverse phase liquid chromatography on an Ultimate 3000 HPLC with 4.6 mm × 250 mm C18 resin column. Samples were resuspended in 120 µl of a solution of 5% acetonitrile and 5% formic acid and loaded onto the column. Samples were eluted as 96 fractions on a gradient ranging from 5 to 35% acetonitrile in 10 mM ammonium bicarbonate. Fractions were concatenated as described previously, and alternating concatenated fractions were lyophilized [19]. Lyophilized fractions were resuspended in a solution of 5% acetonitrile and 5% formic acid prior to analysis by LC–MS.

All mass spectrometry-based analysis was performed on an Orbitrap Fusion Tribrid Mass Spectrometer with in-line Easy nLC System at the University of California San Diego mass spectrometry facility. Samples were loaded onto a column pulled and packed in-house. The inner diameter of the column was 100 µm and the outer diameter was 350 µm. The contents of the column were as follows: the distal tip was packed with 0.5 cm of 5 µm C4 resin followed by 0.5 cm of 3 µm C18 resin. The remaining 29 cm was packed with 1.8 µm C18 packing resin. Peptides were eluted on a 165-min gradient ranging from 11 to 30% acetonitrile in 0.125% formic acid at a flow rate of 300 nl/min. The column was heated to 60 °C.

All data were acquired in centroid mode. Electrospray ionization was achieved through the application of 2000 V of electricity through a T junction connecting sample, waste and column capillaries. MS1 spectra were collected in data-dependent mode using a scan range between 500 and 1200 m/z with resolution of 60,000. Automatic gain control was set to 2 × 10^5^ and the maximum inject time was 100 ms. The top N method for peak selection was selected, with N set to 10 for MS2 and MS3 analysis. Parent ions were selected in the quadrupole at 0.5 Th for MS2 fragmentation. Parent ions were fragmented using collision-induced dissociation (CID) energy and fragment ions were detected in the ion trap with a rapid scan rate automatic gain control of 1 × 10^4^. TMT reporter ion fragmentation was performed using synchronous precursor selection (SPS). The MS2 precursors chosen were fragmented using high-energy collisional dissociation (HCD). Reporter ions were detected in the Orbitrap. The lower limit of detection at the MS3 stage was set to 110 m/z and automatic gain control was set to 1 × 10^5^. The maximum inject time was 100 ms.

### MS data processing

Raw data files were searched using Proteome Discoverer 2.1 using SEQUEST-HT [20]. The reverse database strategy for decoy database generation was used [21–23]. Files were searched against the MAH 104 strain reference proteome. The precursor and fragment ion mass tolerances were set to 50 ppm and 0.6 Da, respectively. The digesting enzyme was specified as trypsin, and up to two missed cleavages were allowed. Peptides of fewer than 6 amino acids or more than 144 amino acids were excluded. A dynamic modification of methionine oxidation (+ 15.995 Da) was included in the search parameters, as were static modifications for isobaric tandem mass tags at the N-termini and on lysine residues (+ 229.163 Da) and carbamidomethylation of cysteine residues (+ 57.021 Da). Filtering of spectra was performed in Percolator at the peptide and protein levels at a 1% FDR (False Discovery Rate) threshold.

Resultant peptide spectral matches (PSM) were manually filtered to exclude spectra without high confidence with (a) rejected PSM ambiguity status, (b) isolation interference larger than 25 and (c) average signal to noise value of less than 10. TMT relative abundance values were summed within proteins matches. Data normalization was performed using a two-step process. TMT signal to noise values were first normalized to the pooled internal standard divided by the median of all internal standard values. The resultant values were then normalized to median signal to noise values for each label divided by the median of all channel median values to account for variable labeling efficiencies.

Volcano plots were generated by comparing treatment with amikacin or clarithromycin to ‘no drug’ treatments within growth conditions (aerobic, anaerobic, and biofilm). A two-tailed *T* test was used to assess significance with Welch’s correction if the variance between samples were unequal. Significance were determined via pi-score to combine the p-value and fold change and rank hits [24] corresponding to an alpha of < 0.05 (i.e. pi score > 1.1082).

The identified MAH proteins were classified into several distinct groups, based on their molecular function. The functional classification was conducted by blasting the amino acid sequence of MAH proteins against the protein sequence database of *M. tuberculosis* strain H37Rv, using the Institute Pasteur’s TubercuList web server (http://genolist.pasteur.fr/TubercuList/). MAH unmatched proteins were classified based on their predicted function or grouped into the hypothetical class.

### Construction of MAH overexpression clones

The MAV_1423 gene, encoding the lipoprotein LprB, was amplified from the MAH genomic DNA (Forward 5′-TTTTTGGATCCA ACGATCCTGATCCCCG-3′ and Reverse 5′-TTTTTGAATTCCATCATCATCATCATCAT TTTCGAGTTCGCAATCGA-3′) and cloned either into the BamH1 and EcoRI restriction sites of the pMV261 mycobacterial shuttle plasmid. The His-tag was incorporated into the reverse primer. In addition, the *lpr*B gene was also cloned into HindIII and HpaI restriction sites of the pMV261 vector containing the Red Fluorescent Protein (RFP) [25] and His-tag at C-terminus using the forward 5′-TTTTTAAGCTTCGATCCTGATCCCC-3′ and reverse 5′-TTTTTGTTAACTTTCGAGTTCGCAAT-3′ primers. The resulted pMV261:LprB and pMV261:LprB:RFP vectors were transformed into MAH competent cells that were prepared by washing bacterial pellet four-times with a chilled wash buffer (10% glycerol and 0.1% Tween-20). The final pellet was resuspended with 1 ml of 10% glycerol. Two hundred µl of competent MAH Cells were electroporated with 7-µl of plasmid DNA using the Gene Pulser Xcell™ Electroporation Systems and kept for 12 h at 37 °C in 7H9 broth. Next, transformed cells were plated on 7H10 agar containing 400 μg/ml of kanamycin. After 14 days of incubation at 37 °C, MAH colonies were screened with PCR for presence kanamycin gene using the following primers: Forward 5′-GTGTTATGAGCCATATTC-3′ and Reverse 5′-TGCCAGTGTTACAACCAA-3′. The PCR program was as follows: 95 °C for 5 min, 35 cycles of 95 °C for 30 s, 57 °C for 30 s, 68 °C for 1 min and a final extension of 68 °C for 5 min. The positive colonies were grown into 7H9 broth supplemented with 400 μg/ml kanamycin.

### The LprB overexpression clone susceptibility to antibiotics

In vitro antibiotic killing assay was performed as described above. Briefly, 3 × 10^5^ cells/ml of MAH104 overexpressing the LprB protein were exposed to 4 μg/ml AMK or 16 μg/ml CLA for 24 h, 48 h, 72 h and 96 h, serially diluted and plated on 7H10 agar plates for CFU counts. The LprB clone growth without antibiotic treatment and MAH 104 clone containing an empty plasmid with and without antibiotic treatment served as controls.

### Biofilm assay

The MAH biofilm formation was examined using the crystal violet staining method as previously described [10]. Approximately, 3 × 10^8^ cells of the pMV261:LprB:RFP overexpression clone and the wild type MAH 104 were resuspended in per ml of HBSS and 300 μl was added into each well of 96-well plate. Plates were incubated at 25 °C in the dark up to 15 days. In addition, to determine if amikacin had enhanced effect on biofilm formation of LprB overexpression clone, 4 μg/ml AMK was added to both the wild type and LprB overexpressing wells. The biofilm formation was quantified at 4, 10 and 15 days by removing supernatant from wells and adding 125 µl of a 1% crystal violet solution for 15 min at room temperature. Next, plates were rinsed four times with HBSS and kept upside down for 30 min. Crystal violet was solubilized in 70 µl of 30% acetic acid at room temperature for 15 min and an optical density was measured at A570 in a plate reader (Epoch Microplate Spectrophotometer, BioTek).

### Macrophage uptake assay using the LprB overexpression clone

Approximately, 3 × 10^5^ THP-1 cells were seeded and differentiated in 24-well plates. Confluent monolayers were infected with MAH clone overexpressing the pMV261:LprB construct at MOI of 10 bacteria:1 cell. After 15 min, 30 min, 1 h and 2 h of infection, THP-1 macrophages were washed three times with HBSS, lysed, serially diluted and plated on 7H10 agar to determine CFUs. MAH carrying the empty pMV261 plasmid was used as a control clone for invasion assay, and the uptake percentage was compared between experimental and control groups at each time point.

### Fluorescence microscopy

Approximately, 200 µl of 3 × 10^8^ bacterial cells/ml containing either pMV261:RFP or pMV261:LprB:RFP vector were inoculated into 8-chamber slides, and biofilms were formed for 7 days at 25 °C. At day 7, wells were fixed with 4% paraformaldehyde and bacterial biofilms were examined using the fluorescent microscope.

### Biofilm matrix preparation

The RFP control and LprB:RFP clones of MAH were incubated for 7 days in HBSS to form biofilms in 75 cm^2^ flasks. Biofilms were extracted as previously described [26]. Briefly, using a sterile swab, biofilms were collected and pelleted by centrifugation at 2500×*g* for 15 min. The biomass pellet was resuspended into 1 ml of HBSS and physically agitated in a Mini-Beadbeater-1 (Biospec Products, Bartlesville, OK) without beads to prevent bacterial lysis. Next, samples were microcentrifuged at 12,000×*g* for 5 min to pellet the bacteria and retain the solubilized matrix in suspension. The supernatants were filtered through a 0.2 μm syringe filter to separate total proteins from the matrix.

### Statistical analysis

Statistical significance for binary comparisons was performed on proteomics data using the Student’s *t* test. The f-test was employed to ensure that the statistical assumption of equal variance required for the Student’s *t* test was met; if it was not, the Student’s *t* test with Welch’s correction was used. Volcano plots were constructed using GraphPad Prism 7. K means clustering was performed on proteomic datasets using the Morpheus K-means clustering tool (https://software.broadinstitute.org/morpheus). The appropriate number of clusters was determined using the elbow method.

Experiments were repeated at least three times, and the results are expressed as a mean ± standard deviation. The comparisons among experimental groups were performed with ANOVA and Student’s *t* test when appropriate. A p value of < 0.05 was considered to be statistically significant.

## Results and discussion

### Delayed killing of MAH by antimicrobials

**Fig. 1:**
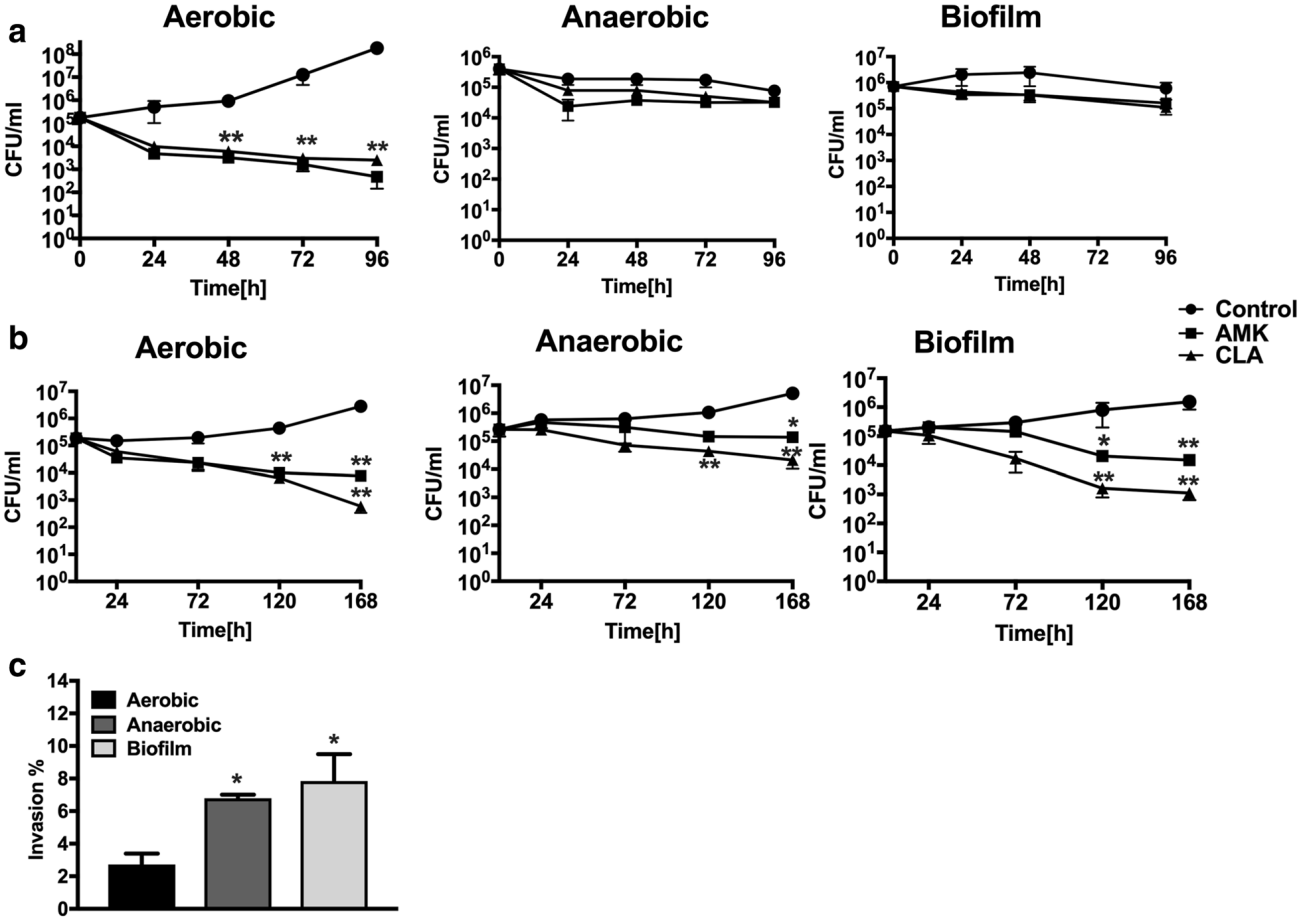
MAH killing dynamics in vitro and in macrophages. **a** MAH 104 time-kill curves of bactericidal concentrations of AMK and CLA show bacterial CFUs over 4 days in aerobic, anaerobic and biofilm conditions. Antimicrobials were added to the 7H9 Middlebrook liquid medium at time zero and bacterial CFUs were compared with the growth control without the drug treatment. **b** MAH104 invasion rates in THP-1 macrophages. Bacteria of the anaerobic and biofilm phenotypes had increased uptake by THP-1 cells when compared to MAH of the aerobic phenotype. MAH were incubated with THP-1 cells for 2 h, extracellular bacteria were removed, and macrophages were lysed for CFU counts. The percentage of invasion was established by calculating the number of bacteria (from inoculum) that entered host cells during 2 h infection. Data are mean ± SD of three independent experiments. *p < 0.05 and **p < 0.01, the significance of differences between MAH104 experimental groups and corresponding bacterial growth controls within the same conditions. **c** MAH104 survival rates in THP-1 macrophages. THP-1 monolayers were infected with bacteria of the aerobic, anaerobic or biofilm phenotype and killing dynamics were recorded over 7 days during AMK or CLA treatment. Bactericidal concentrations of antimicrobials were added to the tissue culture after 2 h infection and then every alternate day. Growth control without drug treatment is also shown. Data are mean ± SD of three independent experiments. *p < 0.05, between MAH aerobic infection rate versus bacterial infection of anaerobic and biofilm phenotype

Using the broth microdilution method, we have determined that the minimal inhibitory concentration (90% of MAH growth inhibition) of AMK and CLA was 1 μg/ml for both compounds; whereas the bactericidal concentrations were 4 μg/ml for amikacin and 16 μg/ml for clarithromycin. The antibiotic killing dynamics were investigated in aerobic, anaerobic and biofilm conditions for 4 days (Fig. 1a). While significant reduction in bacterial viability was observed in the aerobic condition following AMK and CLA exposure over time (p < 0.01 at day 4), only a slight decline in MAH CFU/ml was seen for each treatment group of the anaerobic and biofilm conditions when compared to no antibiotic control.

### MAH killing dynamics by antimicrobials in human macrophages

THP-1 monolayers were infected with MAH expressing either aerobic, anaerobic or biofilm phenotype. For each phenotype, bacterial survival rate within macrophages without antimicrobial treatment served as a control. As shown in the Fig. 1c, THP-1 cells had significantly increased uptake of MAH expressing the anaerobic and biofilm phenotypes when compared with invasion rates of aerobic bacteria (p < 0.05). Furthermore, MAH grown under aerobic conditions had significant killing kinetics in macrophages when exposed to antimicrobials (Fig. 1b); however, even after 7 days of exposure to bactericidal concentrations of antimicrobials, host cells were unable to clear the infection resulting in 2- and 3.5-log decrease of intracellular bacteria during AMK and CLA treatment at day 7, respectively. While MAH obtained from anaerobic condition grew within THP-1 macrophages similarly as the aerobic bacteria, MAH of biofilm phenotype showed a delayed growth initially even without antibiotic treatment, but regained the growth after 3 days. AMK and CLA exhibited killing effect on MAH of both anaerobic and biofilm phenotypes (Fig. 1b), but was considerably lower than drug-killing effects observed in the aerobic condition group. As shown in the Fig. 1b, anaerobic bacteria had its growth maintained intracellularly in presence of antibiotics and showed only 1- or 1.5-log decrease following AMK and CLA treatment at day 7, respectively. In contrast, MAH expressing the biofilm phenotype had 2- and 3-log decrease in intracellular bacterial viability during AMK and CLA treatment at day 7, respectively (Fig. 1b).

### Global proteome response of MAH under aerobic, anaerobic and biofilm conditions and upon exposure to antimicrobials

**Fig. 2:**
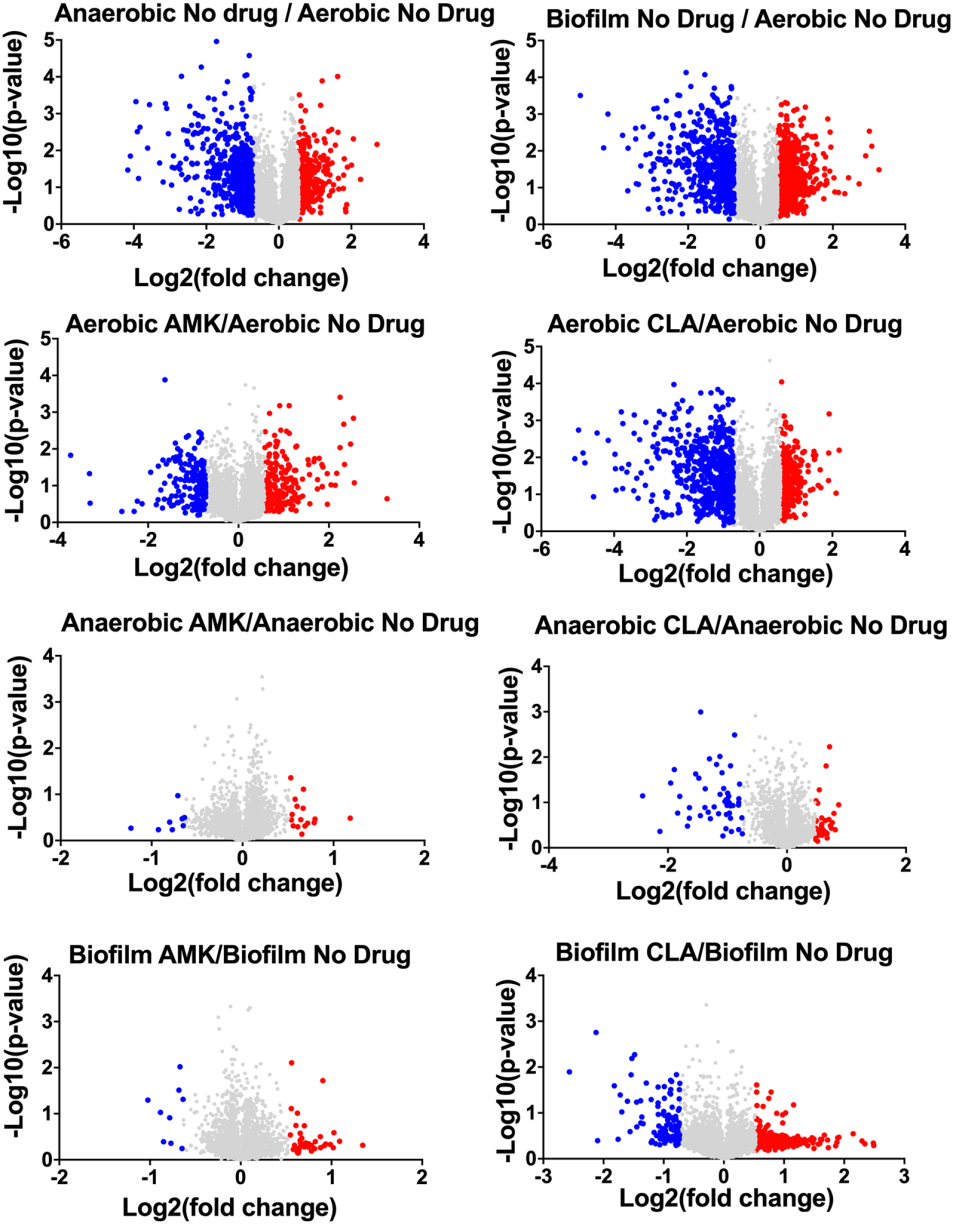
Volcano plots showing proteins that are induced or repressed. Proteins with upregulation ≥ 1.5 fold in red and with downregulation ≤ 1.5 in blue; each dot is one protein. The volcano plot shows the log2-fold changes in gene expression induced by anaerobic and biofilm conditions as compared with aerobic control. The volcano plot also shows the log2-fold changes in gene expression induced by AMK and CLA after 24-h exposure to aerobic, anaerobic and biofilm phenotypes. Each condition is compared with the corresponding condition with no drug control

**Fig. 3:**
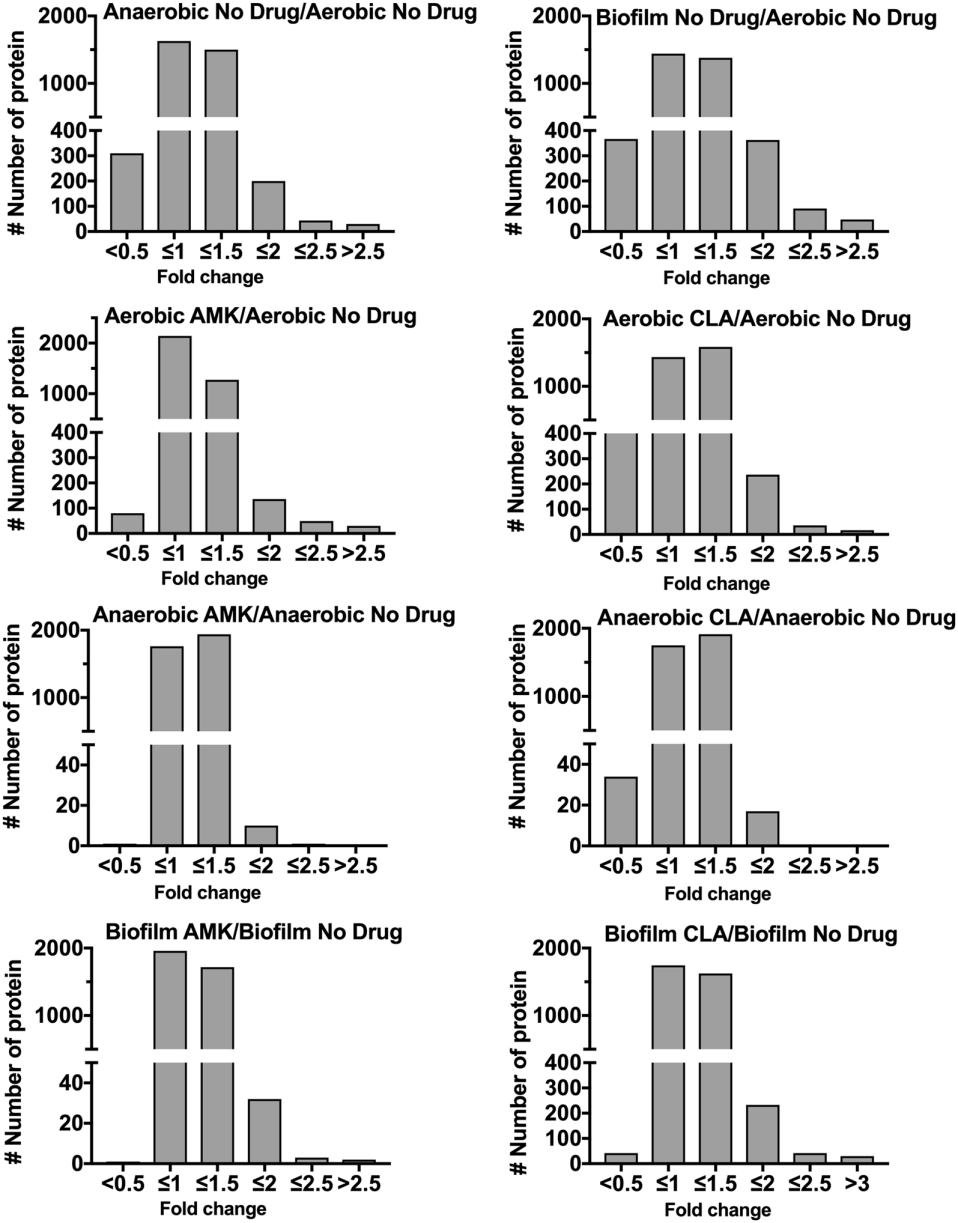
Fold changes of differentially synthesized MAH proteins. The histograms show the distributions of fold changes of proteins enriched under anaerobic and biofilm conditions with and without AMK and CLA treatments

To identify proteomic changes that MAH undergo in conditions encountered within the lung of infected patients and during treatment with antimicrobials, we investigated the pathogen response to aerobic, anaerobic and biofilm conditions with and without AMK or CLA treatment. The quantitative TMT Mass Spectrometric sequencing was performed for samples collected at 24 h time point and, overall, 4000 proteins were identified across all experimental and control groups. The identified protein list along with their normalized spectral counts, annotations and fold changes over control at corresponding time points for each condition and antimicrobial is presented in the Additional file 1. Volcanic plots, presented in the Fig. 2, gives a global overview of induced and repressed proteins following bacterial exposure to different environmental conditions and antibiotics. More specifically, while the incubation under anaerobic and biofilm conditions resulted in enrichment of 409 and 603 proteins, the synthesis of 522 and 585 proteins were downregulated in MAH under aerobic condition. Proteome analysis of MAH treated with AMK for 24 h revealed 263, 17, 41 enriched and 178, 8, 9 downregulated proteins in aerobic, anaerobic and biofilm conditions, respectively, when compared to control, a no drug treatment group. In presence of CLA, 379, 28, 380 proteins were upregulated and 598, 50, and 107 proteins were downregulated in aerobic, anaerobic and biofilm conditions, respectively, when compared with only control conditions. Histograms of the Fig. 3 demonstrate the distribution of the average fold changes for proteins at different environment conditions with or without antibiotic treatment.

**Fig. 4:**
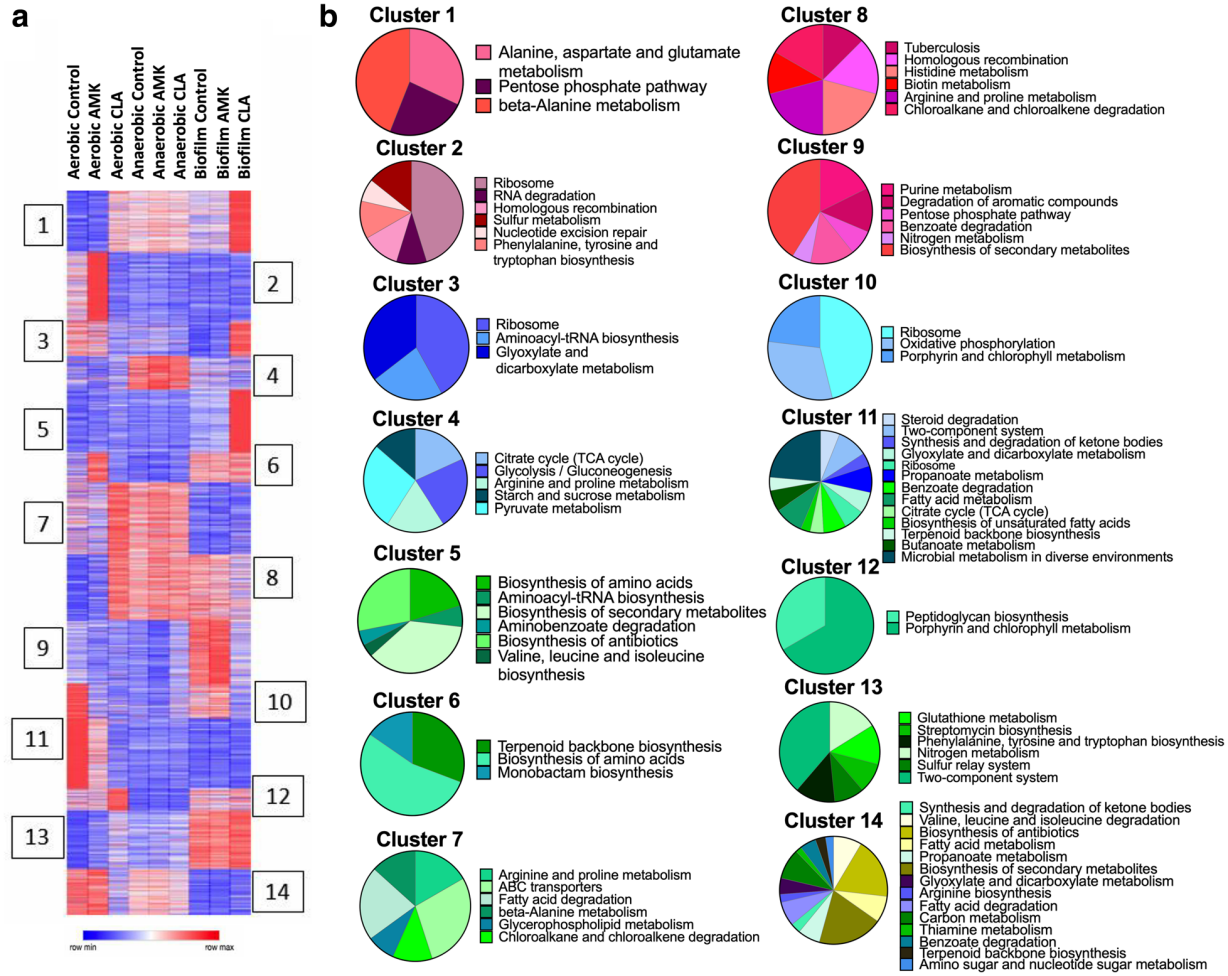
The heatmap and clustering analysis for metabolic pathways. **a** Heatmap of K-means clustering have identified groups of proteins with various expression in different treatment groups. The heat map color codes reflect a change. K-means clustering was performed for 3963 protein-coding genes across all studied groups (adjusted p-value < 0.05). The model-based optimal number of K = 14 was determined. **b** Fourteen Pie charts present the KEGG enrichment analysis for metabolic pathways and correspond to 14 clusters

For a global overview of proteomic changes in MAH response to conditions and antibiotics, without exclusively focusing on the induced proteins only, we used Morpheus to identify K-means clustering (Fig. 4a). We compared protein levels in each drug treatment versus untreated bacteria using an analysis paired by antibiotic and condition. In addition, with the elbow method we found 14 optimal number of clusters. The heat map shows that proteins of 1 and 5 clusters are mainly produced under biofilm formation when exposed to clarithromycin for 24 h. In contrast, proteins of 2, 7, 11 and 14 clusters are downregulated in biofilms with and without AMK and CLA treatment, proteins clustered in 4, 7 and 8 groups are highly upregulated in the anaerobic condition with and without AMK and CLA treatment. Pie charts in the Fig. 4b demonstrate metabolic pathway enrichment related to each cluster.

### Functional grouping of 1.5-fold and more synthesized MAH proteins

**Fig. 5:**
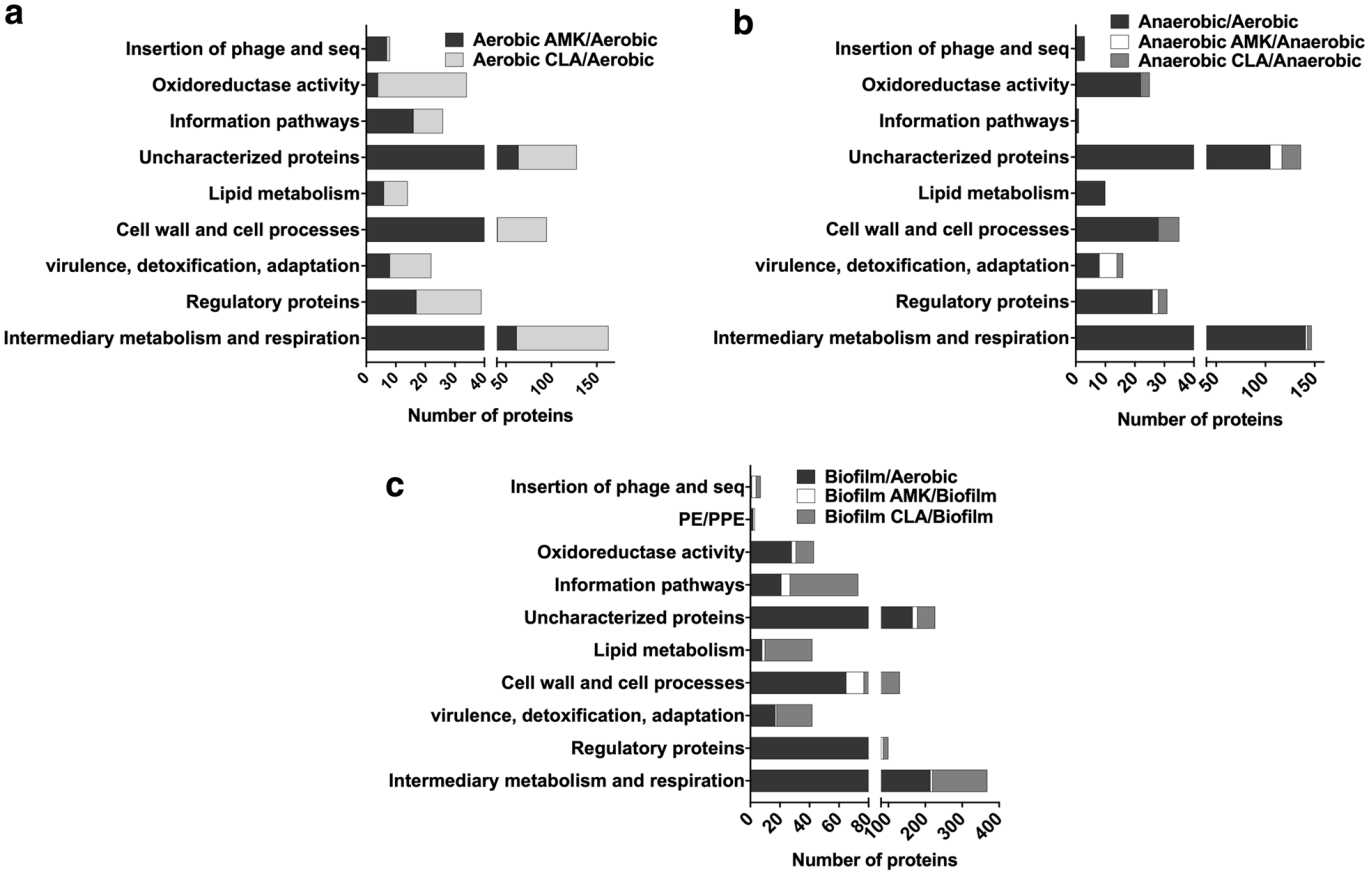
Functional classification of MAH proteins synthesized 1.5-fold and more was done by finding the protein homologs of H37Rv strain of *Mycobacterium tuberculosis* and using the functional categorization available on TubercuList webserver of Institute Pasteur. MAH proteins that did not match to any proteins of H37Rv strain were classified based on predicted or conserved domain/motif search at NCBI (National Center for Biotechnology Information) and the Pfam database through the Sanger Institute. Histograms show number of proteins belonging to (**a**) aerobic, (**b**) anaerobic or (**c**) biofilm functional category with and without antibiotic treatment

Mass spectrometric analysis of MAH104 samples found a total of 225 proteins and 304 proteins synthesized 1.5-fold and more during 24 h amikacin and clarithromycin exposure in the aerobic condition. We categorized differentially synthesized MAH proteins into functional groups by blasting the amino acid sequences of found MAH proteins against the protein sequence database of well-studied *M. tuberculosis* strain H37Rv on the Institute Pasteur’s TubercuList web server. MAH proteins that did not show any homologies to *M. tuberculosis* genome were classified based on their predicted function or grouped into the hypothetical class. Database search for domain/motif analysis was performed using the BLAST network service available through the National Center for Biotechnology Information and the Pfam database through the Sanger Institute. Most represented categories in the aerobic condition during MAH exposure to both antibiotics were intermediate metabolism and respiration (163 proteins), cell wall and cell processes (95 proteins), regulatory proteins (39 proteins), metabolic enzymes falling into oxidoreductase activity category (34 proteins), information pathway (26 proteins), virulence, detoxification, adaptation (22 proteins), and majority of proteins with unknown function (128 proteins) (Fig. 5a).

While 344 proteins were synthesized with 1.5-fold enrichment and more in the anaerobic condition, exposure to AMK and CLA for 24 h induced 23 and 38 protein synthesis, respectively. The distribution of represented categories was the following: 141, 2 and 2 proteins of the intermediate metabolism and respiration, 26, 2 and 2 of regulatory proteins, 8, 6 and 2 proteins from the virulence, detoxification, adaptation group, and enzymes with the oxidoreductase activity were 22, 0 and 3 proteins in the anaerobic-no drug, anaerobic-AMK and anaerobic-CLA groups, respectively (Fig. 5b).

During biofilm formation, MAH synthesized 603 proteins with 1.5-fold and more. The treatment with AMK and CLA of MAH biofilms resulted in the synthesis of 53 and 380 proteins, respectively. The distribution of represented categories for biofilm-no drug, biofilm-AMK and biofilm-CLA experimental groups were the following: intermediary metabolism and respiration (214, 6 and 148 proteins), regulatory proteins (81, 6 and 13 proteins), in virulence, detoxification, adaptation (17, 1 and 24 proteins), cell wall and cell processes (65, 12 and 54 proteins), lipid metabolism (8, 2 and 32 proteins), information pathways (21, 6 and 46 proteins), proteins with oxidoreductase activity (28, 3 and 12 proteins), and majority of uncharacterized proteins (166, 13 and 48 proteins) (Fig. 5c).

**Fig. 6:**
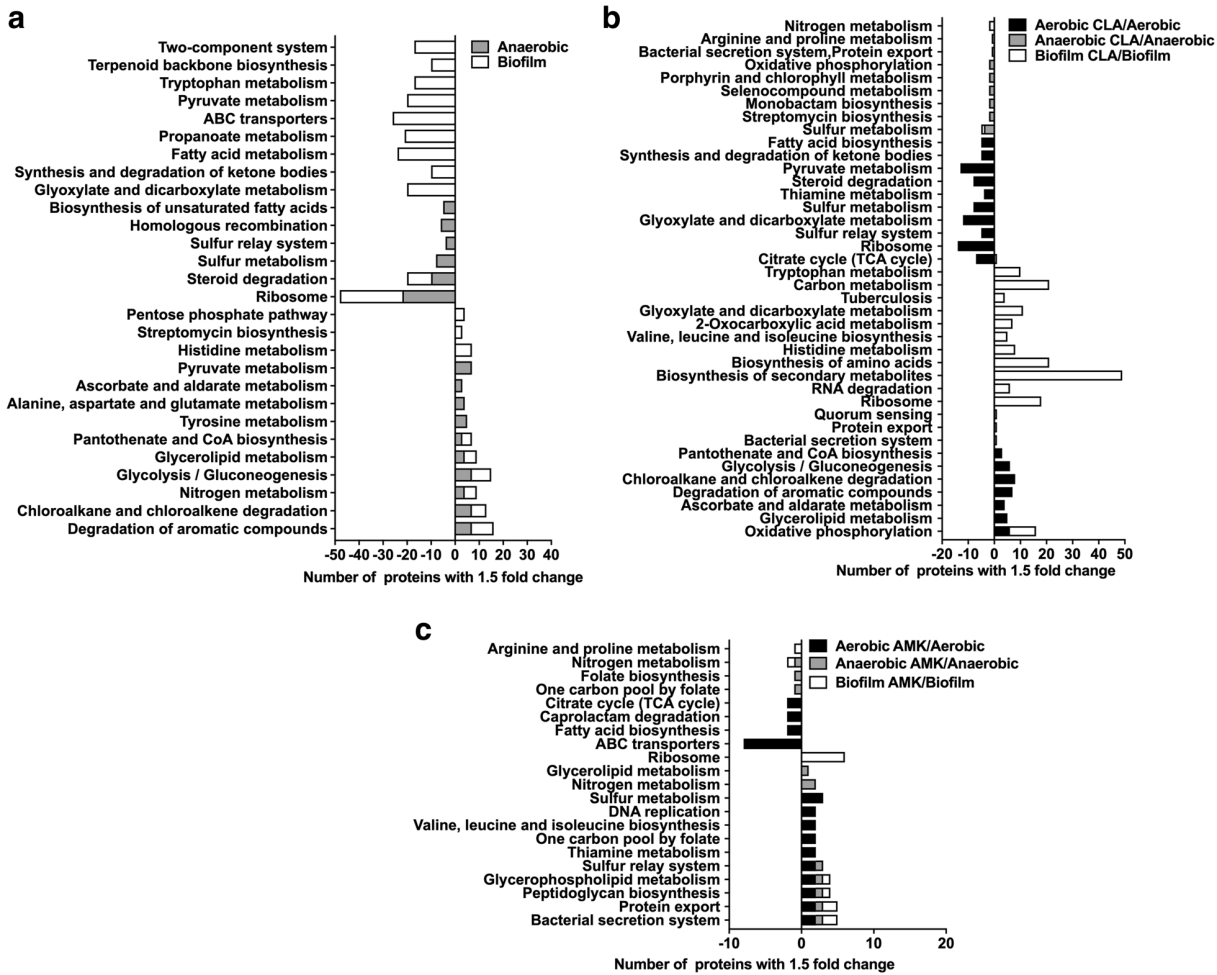
MAH metabolic pathway enrichment. Proteins are grouped based on the KEGG pathway database for differentially synthesized proteins in **a** anaerobic and biofilm, **b** CLA treatment aerobic, anaerobic and biofilm, and **c** AMK treatment aerobic, anaerobic and biofilm groups

In addition, the metabolic pathway enrichment in condition and drug treatment groups are presented in Fig. 6. The protein assignment to KEGG metabolic pathways showed several over-represented metabolic categories that are further discussed below.

### Characterization of MAH metabolic pathways expressed under environmental conditions

**Fig. 7:**
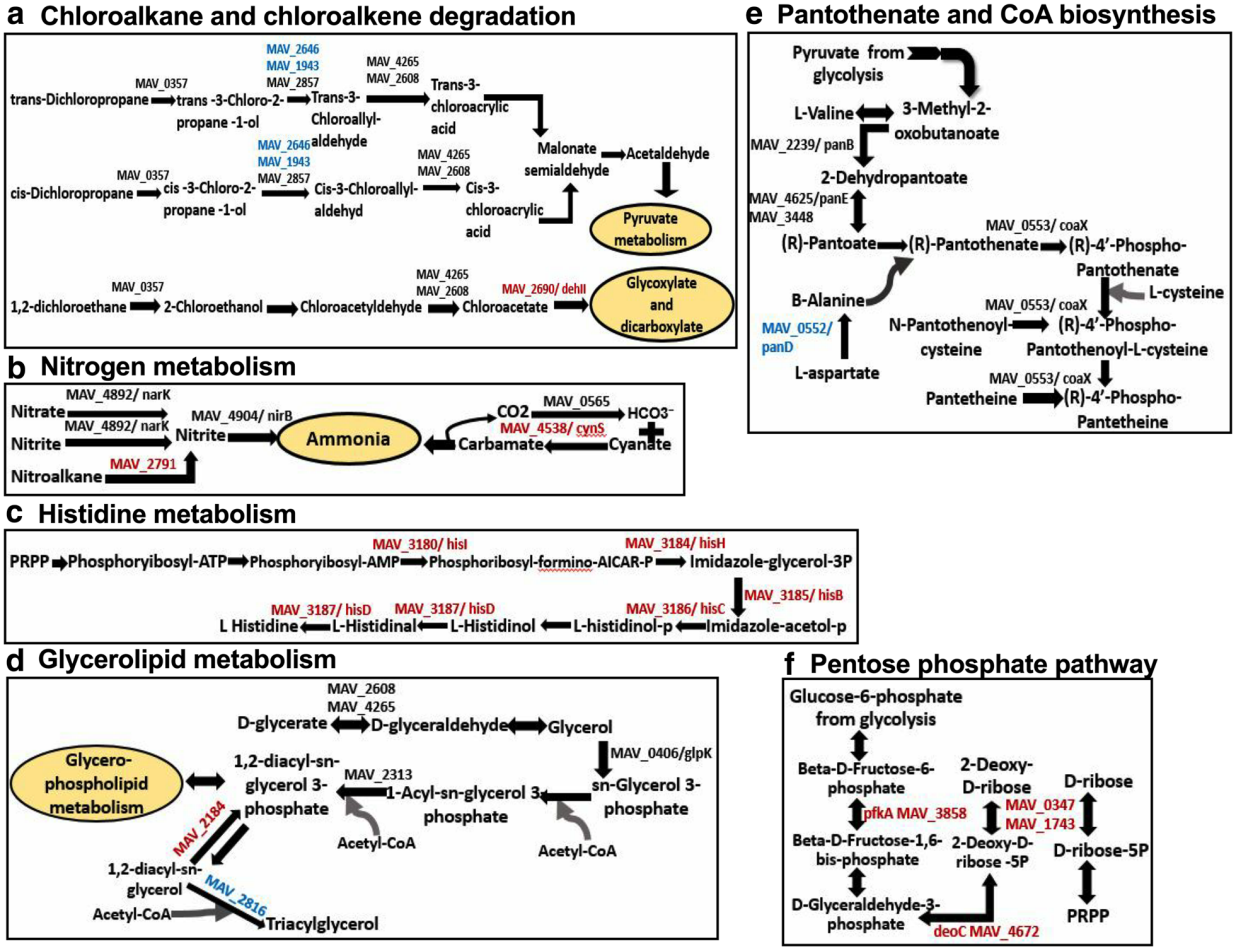
Upregulated metabolic pathways of MAH in anaerobic and biofilm conditions. Bacterial proteins synthesized during biofilm formation are presented in red, during anaerobic condition in blue and in both conditions in black

Ten metabolic pathways upon expression of the anaerobic phenotype and nine pathways of the biofilm condition were identified to show significant expression when compared with the aerobic condition alone (Fig. 6a). Among them six pathways were common between anaerobic and biofilm conditions, including the majority of highly synthesized enzymes of chloroalkane and chloroalkene degradation pathways (Fig. 7a). The chloroalkane pathway leads to the acetaldehyde production, which reversibly can be converted into an acetyl-CoA, acetyl-phosphate and pyruvate, and then processed in the TCA cycle for ATP synthesis. On the other hand, the chloroalkene degradation produces substrate for the glyoxylate shunt, which is a modified Krebs cycle and occurs in mycobacteria [27]. The glycoxylate cycle has a central role in the metabolism of pathogenic mycobacteria and, therefore, enzymes associated with the glycoxylate shunt has been exploited for the development of additional anti-TB therapy [28–31]. Because the isocitrate lyase (ICL) converts the isocitrate into glyoxylate and malate synthase, which in turn catalyzes the conversion of malate from glyoxylate, ICL has been proposed to be a potential target for the latent tuberculosis [29, 30]. In addition, some of the glyoxylate could be diverted into the reductive amination pathway to produce NAD from its reduced form [32]. In fact, reductive amination of the glyoxylate by glycine dehydrogenase has been demonstrated to be an alternative energy source for *M. tuberculosis* during nonreplicative state and aids the pathogen to tolerate anaerobic conditions [33, 34].

While our study identified nitrate, nitrite transporter and nitrite reductase enzymes of MAH highly upregulated under anaerobic and biofilm conditions (Fig. 7b), in *M. tuberculosis* research, it has been extensively documented that an increased nitrate use had a high impact on bacterial survival during hypoxia by replacing oxygen as terminal electron acceptor [35, 36].

Furthermore, our data shows that histidine metabolism pathway was upregulated in MAH biofilm when compared with bacteria of the aerobic phenotype (Fig. 7c). It is known that the histidine metabolism is an important pathway promoting bacterial biofilm formation [37–39]. A proteomic study of *Acinetobacter baumannii* biofilms and functional analysis using the gene knockout mutants reveled several cell surface proteins implicated in biofilm formation were also associated with the histidine metabolism [40]. In *Staphylococcus xylosus* research, it has been demonstrated that histidine metabolism has a role on the *S. xylosus* biofilm formation, also IGPD enzyme (imidazoleglycerol-phosphate dehydratase) involved in histidine metabolism played a crucial role in a fourth-generation cephalosporin (cefquinome) inhibition of biofilm formation [41, 42]. In addition, while both auxotrophic and wild-type strains of *M. tuberculosis* are able to survive prolonged starvation, the histidine auxotroph clone is unable to survive a single-amino-acid starvation [43]. In our study, we found that imidazoleglycerol-phosphate dehydratase (hisB/MAV_3185) as well as other enzymes (hisD/MAV_3187, hisC/MAV_3186, hisI/MAV_3180, hisH/MAV_3184) related to the histidine metabolism were highly synthesized under biofilm conditions and raises the possibility that histidine may play an important role during MAH biofilm formation and under oxygen starvation either by be converted into other biological active amines or converted to 4-imidazolone-5-propionate in a sequence of reactions resulting in formation of glutamate and ammonia.

Another pathway upregulated in MAH under the anaerobic and biofilm conditions is glycerophopholipid metabolism. As shown in the Fig. 7d, the glycerol and fatty acids are used to form 1, 2-diacyl-sn-glycerol and is processed by MAV_2184 into 1, 2-diacyl-sn-glycerol 3-phosphate during biofilm formation. In contrast, MAV_2816 converts 1, 2 diacyl-sn-glycerol 3-phosphate to triacylglycerol in MAH under anaerobic condition.

Several enzymes of pantothenate and CoA biosynthesis pathways were upregulated in both anaerobic and biofilm conditions (Fig. 7e). Pantothenate (vitamin B5) is a precursor for the biosynthesis of CoA, a cofactor involved in tricarboxylic acid cycle, phospholipids synthesis, fatty acids synthesis and degradation [44]. The flux of carbon through Acetyl-CoA is difficult during non-replicating condition of MAH. Acetyl-CoA is synthesized from fatty acid degradation and uses the glyoxylate shunt to provide the carbon for carbohydrate synthesis [45]. It has been shown that the *panC* and *panD* genes of MAH and *M. tuberculosis* are involved in de novo biosynthesis of pantothenate [46]. By infecting immunocompromised SCID mice with *M. tuberculosis* ΔpanCD knockout mutants, it has been observed that animals survived for more than 36 weeks; whereas *M. tuberculosis* WT infected mice survived for only 5 weeks [47].

The proteome analysis identified the pentose phosphate pathway highly upregulated only in MAH biofilms (Fig. 7f). In the pentose phosphate pathway, the glucose turnover process leads to the synthesis of NADPH and pentose, essential parts of nucleotides. This pathway also produces the phosphoribosyl pyrophosphate (PRPP) from ribose-5P, which is used in the biosynthesis of histidine and purine/pyrimidine [48]. As highlighted above, the histidine metabolism appears to play a very important role in maintaining of bacterial biofilms.

### MAH metabolic pathways expressed under different environmental conditions in presence of antibiotics

**Fig. 8:**
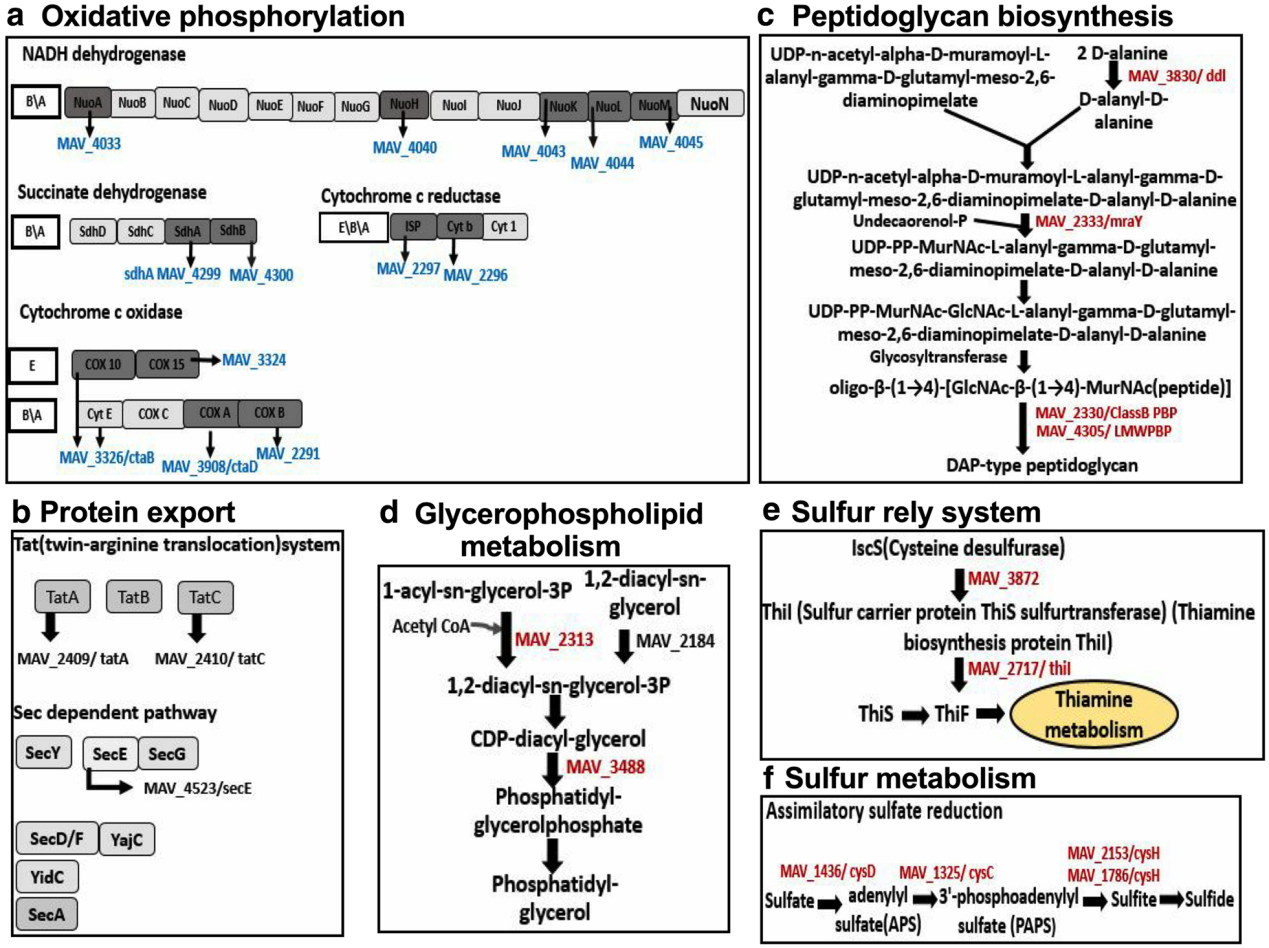
Upregulated metabolic pathways of MAH in aerobic, anaerobic and biofilm conditions when exposed to bactericidal concentrations of amikacin and clarithromycin for 24 h. Bacterial proteins synthesized in all tested conditions during CLA treatment are presented in blue, during AMK treatment in red and in both drug-groups in black

We identified seven metabolic pathways expressed under aerobic condition and twelve under biofilm condition when treated with CLA, and the expression of these pathways were significantly greater than the one seen in the aerobic condition alone (Fig. 6b). The oxidative phosphorylation pathway was more prominent in both aerobic and biofilm conditions during presence of CLA with total of twelve proteins synthesized by several-fold higher than in the control group (Fig. 8a). While oxygen is the final electron acceptor in aerobic condition, nitrate or fumarate could be used as an electron acceptor in anaerobic conditions [49]. Electron transport in mycobacteria is initiated by NADH dehydrogenases (NDH), succinate dehydrogenases (SDH) and various cytochrome oxidases [50]. Proteins nuoAHKLM (MAV_4033, MAV_4040, MAV_4043, MAV_4044 and MAV_4045) identified in this study belong to the NADH dehydrogenase group; whereas MAV_4300 and sdhA (MAV_4299) are succinate dehydrogenase enzymes and facilitate electron transport in MAH. The succinate dehydrogenase enzymes predominantly act in the citric acid cycle to oxidize succinate to fumarate. Our results demonstrate that, in presence of clarithromycin, the TCA cycle is downregulated and oxidative phosphorylation is upregulated. Increased oxidative phosphorylation in mycobacterial leads to generation of increased transmembrane proton gradient through electron transport chain (ETC) and constitutes the proton motive force (PMF). As a result, protons that translocate through ETC are used by efflux pumps (EPs) to extrude drugs [51]. Thus, in aerobic and biofilm conditions MAH can export clarithromycin. In addition, cytochrome C oxidase and reductases has been shown to regulate this process, and our results identify several proteins related to the cytochrome C oxidase process [51, 52].

Other notably upregulated pathways under anaerobic condition during the presence of clarithromycin are bacterial Sec and Tat secretion systems and quorum sensing related protein MAV_4523 (protein translocase subunit SecE) (Fig. 8b). The twin-arginine translocation (Tat) system is comprised of three membrane proteins of TatA, TatB and TatC actively involved in transport of unfolded proteins from the cytoplasm to exterior of the cell. TatA (MAV_2409) and TatC (MAV_2410) proteins are also greater synthesized in MAH of aerobic phenotype during exposure to both amikacin and clarithromycin separately over the aerobic control. Higher level of TatA protein synthesis is triggered by AMK in MAH during biofilm formation as well when compared to the control no treatment. In addition, the mycobacterial translocation channel SecE (MAV_4523) was identified in biofilm and anaerobic tested groups of MAH exposed to AMK for 24 h.

As summarized in the Fig. 6c, AMK exposure to MAH of aerobic, anaerobic and biofilm phenotypes lead to upregulation of ten, seven and five metabolic pathways, respectively. The glycerophospholipid, peptidoglycan biosynthesis, bacterial secretion and protein export systems were among common metabolic pathways upregulated in AMK treated experimental group under all three conditions. Penicillin-binding proteins (PBPs) belong to the peptidoglycan biosynthesis pathway and are associated with the final stage of bacterial cell wall assembly (Fig. 8c). The carboxypeptidase enzyme MAV_4305, a low molecular weight PBP, moderates the degree of cross-linking by removing the terminal d-Ala from the peptidoglycan. The enzyme MAV_4305 can also produce Lys-type peptidoglycan. While MAV_4305 was highly upregulated in biofilms, MAV_3830 (d-alanine–d-alanine ligase) and MAV 2333 (Phospho-N-acetylmuramoyl-pentapeptide-transferase) enzymes had increased expression under aerobic condition during AMK treatment.

The potential changes in structure and hydrophobic properties of the cell wall produce new permeability barrier that can increase tolerance to antibiotics [53, 54]. The mycobacterial cell plasma membrane consists of Phosphatidylethanolamine (PE), phosphatidylserine (PS), phosphatidylinositol (PI), phosphatidylglycerol (PG) and cardiolipin (CL). Deficiency in any of these components of the cell wall effect bacterial susceptibility to antibiotics [55]. MAV_3488, MAV_2184 and MAV_2313 proteins, highly upregulated in AMK treatment group, belong to the enzyme group that is involved in the glycerophospholipid metabolism (Fig. 8d) and most likely paly role in MAH phenotypic resistance to drugs.

Another prominent metabolic pathway, highly upregulated in aerobic and anaerobic bacteria and triggered by AMK treatment, was the sulfur relay system (Fig. 8e). A sulfur transfer is required step for synthesis of cofactors of molybdenum and thiamin, and is carried by unique sulfur carrier proteins MoaD and ThiS activated through adenylation by E1-like enzymes [56, 57]. In this study, cysteine desulfurase (IscS/MAV_3872) was upregulated to donate sulfur for molybodenum cofactor biosynthesis. It has been demonstrated in *Escherichia coli* that IscS converts the MoaD acyl-adenylate into a thiocarboxylate during molybdenum cofactor biosynthesis and supports molybdopterin synthesis [58]. The IscS enzyme has the central role in donating the sulfur to FeS clusters, the thionucleosides 4-thiouridine and 5-methyl aminomethyl-2-thiouridine and thiamin [59, 60].

Furthermore, proteins of sulfur metabolism, specifically, sulfur assimilation were cysH/MAV_1786 and cysH/MAV_2153 phosphoadenosine phosphosulfate reductase enzymes were upregulated in MAH of aerobic condition during AMK treatment (Fig. 8f). The phosphosulfate reductase enzymes convert an adenylyl sulfate (APS) to phosphoadenosine phosphosulfate (PAPS); whereas PAPS is the universal sulfate donor for sulfotransferases [61]. Sulfide is a final product of sulfur metabolism and is used in biosynthesis of coenzymes, S-containing amino acids such as cysteine and methionine, and mycothiol. Thiol containing small detoxifying molecule mycothiol (MSH) removes numerous bactericidal agents to maintain the highly reducing environment within bacteria and protect cells against disulfide stress [48]. We identified the alkanesulfonate monooxygenase (MAV_3426), which is involved in converting alkane sulfonate and methane sulfonate into sulfite and was upregulated in MAH of aerobic phenotype due to AMK treatment. It has been demonstrated that *M. tuberculosis* sulfur metabolism genes, including cysH transcriptional regulator, are highly upregulated in nutrient deprived conditions, hypoxia and sulfite assimilation increases due to exposure to antibiotics [57, 62].

### Characterization of LprB lipoprotein

**Fig. 9:**
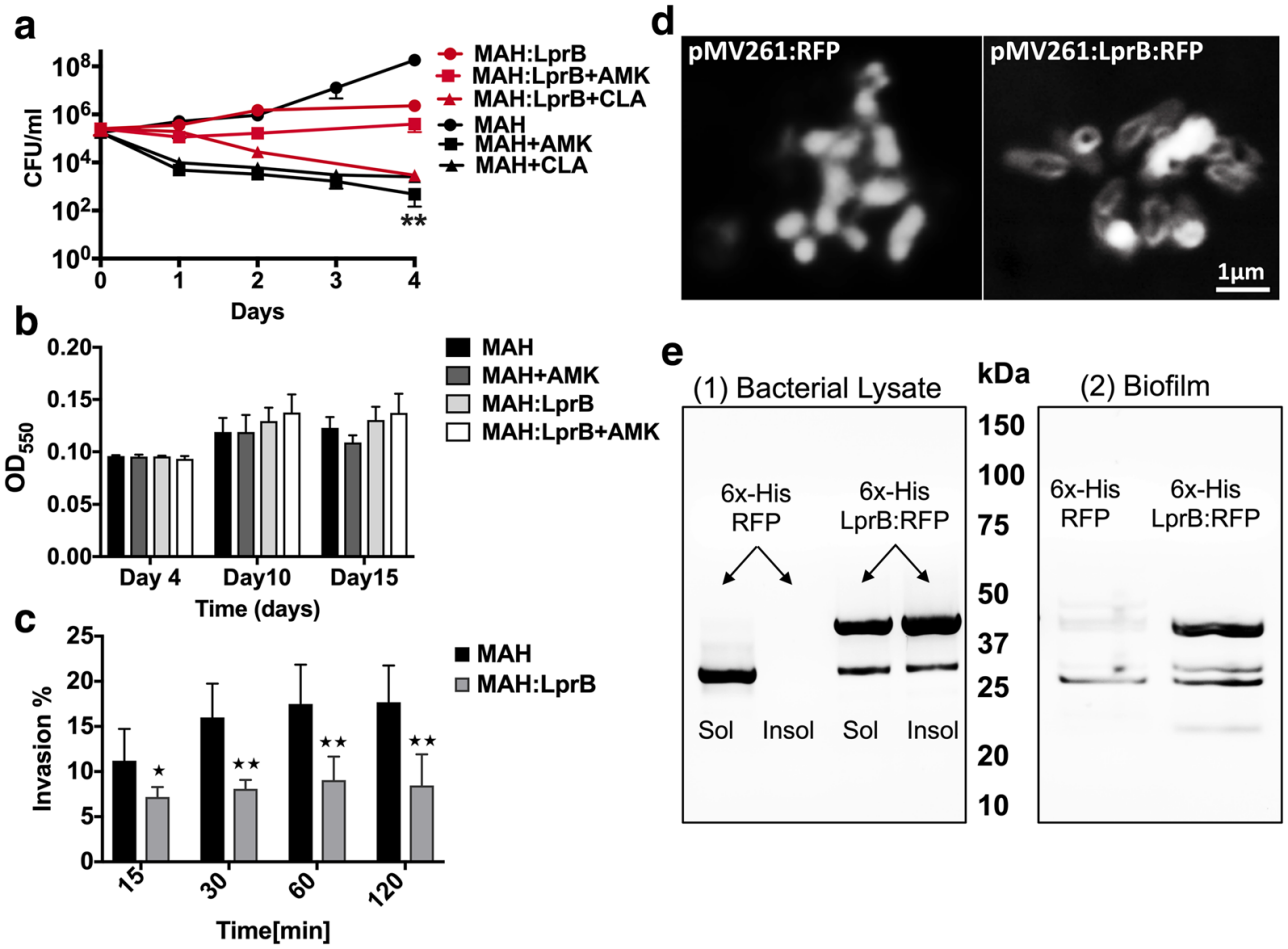
Effects of LprB overexpression on MAH tolerance to drugs and biofilm formation**. a** In vitro time-kill curves for MAH LprB overexpressed clone with and without exposure to bactericidal concentrations of AMK and CLA over 4 days. Results represent means ± standard errors of three independent experiments. **b** MAH control and LprB overexpression clones (3 × 10^8^ CFU/ml) were inoculated in PVC 96-well plates with HBSS for 15 days in presence or absence of AMK. The biofilm was evaluated using crystal violet stain as described in Materials and Methods. The A570 readings from three experiments are shown and values are means ± standard deviations. **c** The time dependent phagocytosis assay of MAH by THP-1 macrophages shows significant differences between the uptake of LprB overexpression clone and the wild type control over time. The experiment was performed in triplicate, repeated three times and means ± SDs were determined. The significance between control and experimental groups is **p < 0.01. **d** The visualization of RFP fusion LprB protein by fluorescent microscopy, in contrast to MAH control expressing only RFP. **e** Western blot analysis of soluble and insoluble fractions of MAH expressing 6×-His tagged proteins. Soluble proteins were first extracted by mechanical disruption in HBSS. Insoluble proteins were then extracted in a denaturing urea solution. Cell envelope and membrane localizing proteins tend to remain insoluble in HBSS, while cytoplasmic and secreted proteins are readily extracted in HBSS. (1) Cell fractionation shows that LprB protein is found in the insoluble and soluble fraction of MAH, while RFP protein remains only in soluble portion. (2) Biofilms were formed in HBSS at 37 °C for 7 days. Supernatants were gently removed and total proteins of the biofilm biomass were extracted in HBSS. In contrast to MAH control expressing only RFP, we observe that LprB lipoprotein is translocated into the MAH biofilm matrix

We hypothesized that MAH proteins that are commonly synthesized within different environmental stresses and following incubation with bactericidal concentrations of antibiotics most likely belong to cellular pathways that promote bacterial persistence and are associated with a tolerance phenotype of MAH. To test the concept, we selected MAH lipoprotein LprB synthesized 1.87- and 3.63-fold greater in CLA and AMK treated experimental groups and 1.3-fold higher in the anaerobic condition than in the MAH of biofilm phenotype. Due to the fact that *M. tuberculosis* LprB has been demonstrated to be essential for virulence and survival in vivo, we overexpressed the *lpr*B gene in MAH using the pMV261 vector under hsp60 constitutive promoter. The LprB protein overexpressed clone was tested for in vitro antibiotic killing kinetics, biofilm formation assay and for the ability to invade human macrophages (Fig. 9). The killing curves for MAH control and LprB experimental clone were established for over 4 days of exposure to AMK and CLA. In vitro bacterial killing kinetics for 3x10^5^ bacteria/ml shows increased resistance to amikacin and clarithromycin when compared to the MAH control groups (Fig. 9a). While both antibiotics had significant bactericidal activity against the wild type and decreasing MAH viability with 5-log by CLA and 6-log by AMK, the overexpression of LprB resulted in bacterial tolerance and decreased MAH viability only 0.5-fold in AMK and 2-log in CLA treatment groups. We did not observe any significant changes in biofilm formation between MAH wild type and LprB clone either in presence or absence of amikacin (Fig. 9b). In addition, our results demonstrate that by overexpressing the LprB in MAH104, the pathogen is not phagocytosized by human macrophages as effectively as the wild type strain containing the empty plasmid. The time dependent invasion assay shows significant differences between the uptake of LprB and the wild type control over time (Fig. 9d).

Using the fluorescent RFP control construct of MAH and pMV261:LprB:RFP overexpressing clone, we observe that LprB protein localizes to the bacterial cell surface (Fig. 9d) and is found in the soluble and insoluble fraction of MAH (Fig. 9e(1)). Bacteria expressing recombinant protein were mechanically disrupted and soluble total protein fraction was extracted. Protein remaining in the insoluble fraction was extracted in a denaturing buffer containing urea. Protein fractions were separated via SDS-PAGE and analyzed by Western blot to determine the solubility of the RFP and LprB protein constructs. The results indicate that the full length LprB:RFP is accessible from the soluble and insoluble fractions, whereas RFP control localizes primarily to the soluble fraction. In addition, by extracting and analyzing the biofilm biomass for LprB presence busing the western blotting, we demonstrate that MAH lipoprotein localizes into the biofilm matrix (Fig. 9e(2)).

## Conclusions

*Mycobacterium avium* subsp *hominissuis* is the most common intracellular pathogen associated with disseminated infection in patients with HIV/AIDS [12]. Individuals with pre-existing chronic lung diseases, such as cystic fibrosis, bronchiectasy and emphysema, and patients who are receiving immunosuppressive therapy, are also predisposed to pulmonary MAH infection [2]. Despite recent advancements in the discovery of new anti-mycobacterial compounds and development of novel delivery methods to quickly achieve the bactericidal concentrations in infected sites and tissues, it is still major challenge to efficiently eliminate infecting organism [6]. The action of antibiotics against mycobacteria is more complex than initially thought. Recent studies on *M. tuberculosis* and on MAH (this study) have shown that although antibiotics are employed at bactericidal concentrations, aiming to kill the pathogen, bacteria viability is still not affected in a significant manner for several days [63].

The adherence to the lengthy therapy is a crucial component for treatment of diseases caused by mycobacterial pathogens. The reasons for the extended treatment are complex and reflect the inability of current antimicrobials to clear phenotypic heterogeneous bacteria quickly, particularly, the subpopulation of susceptible but drug-tolerant bacilli where the persistent fitness to anti-mycobacterial drugs is in fact stimulated and enhanced by the host environmental stresses. In order to enhance MAH killing and eliminate bacteria in different metabolic states (drug-tolerance phenotype) where current antimicrobials are ineffective, a rational therapy needs to be developed. This strategy aims to: (1) identify global proteomic remodeling of MAH within the specific niche of different environmental conditions of the host with and without antibiotic treatments, (2) determine and then (3) target significant bacterial pathways associated with the MAH tolerant/persistent phenotype.

It is widely accepted hypothesis that in order to overcome unfavorable environmental conditions and killing effect of antimicrobials, bacteria require expression and synthesis of specific subsets of proteins that play important role in adaptation within new conditions and, consequently, contributing to a long-term survival of the pathogen. To test this hypothesis, we investigated the relative protein quantitative analysis of MAH proteome under aerobic, anaerobic and biofilm conditions with and without exposure to antimicrobials using Tandem Mass Tag Mass Spectrometry approach. Results obtained suggest that under conditions encountered in the lung, MAH activates several alternative pathways that can utilize mucin, phospholipids, nitrates, glucose and amino acids such as histidine to support its metabolic activities. All of these substrates, in general, are largely present in the rich environment of the diseased lung. Bacterial metabolic shift in response to the action of antibiotics including synthesis of protective peptidoglycan, oxidative posphirylation, and electron transfer are most likely the pathogen’s attempt to support and maintain key metabolic pathways highly effective. MAH also activates the protein export pathway, which suggests that the pathogen may transport effector molecules on the cell wall and contribute to bacterial tolerance within new environments. The identified cellular processes are widely demonstrated in the literature to be associated with mycobacterial metabolic shift and, subsequently, promoting drug-tolerance state and survival. In addition, the regulation of certain virulence related factor such as LprB associated with aminoglycoside resistance and decreased macrophage uptake can become a possible anti-MAH target.

The phenotypic changes induced by environmental conditions as well as by the presence of antibiotics may have an impact in the ability of the host to defend against the pathogen. Our findings reported here add important information on MAH response to therapy, filling some of the gap on the basic understanding how this pathogen respond to different host environments and drug treatments.

## Supplementary information

**Additional file 1.** MAH proteins obtained by the quantitative TMT mass spectrometric sequencing for each condition and antimicrobial is attached as the XLSX file.

## Abbreviations

MAH: *Mycobacterium avium* subsp*. hominissuis*
NTM: nontuberculous mycobacteria
CFU: colony forming unit
AMK: amikacin
CLA: clarithromycin
MIC: minimal inhibitory concentration
HBSS: Hank’s Balanced Salt Solution
EDTA: ethylenediamine tetraacetic acid
IAA: iodoacetamide
DTT: dithiothreitol
SDS: sodium dodecyl sulfate
HEPES: 4-(2-hydroxyethyl)-1-piperazineethanesulfonic acid
TCA: trichloroacetic acid
TMT: tandem mass tag
CID: collision-induced dissociation
SPS: synchronous precursor selection
HCD: high-energy collisional dissociation

## Availability of data and materials

Please contact author for data requests.
